# Conjunctival changes and inflammatory aspects in rabbits' conjunctivas induced by fixed combinations of prostaglandin analogues and timolol maleate

**DOI:** 10.1186/1869-5760-3-22

**Published:** 2013-01-28

**Authors:** Nubia Vanessa Lima de Faria, Heloisa Helena Russ, Palloma Rose, Lucia Noronha, Paulo Augusto Mello, Fabiano Montiani-Ferreira, Sebastião Cronemberger Sobrinho

**Affiliations:** 1 Ophthalmology Department, Federal University of Minas Gerais, Av. Alfredo Balena, 190 3rd floor room 3005, Belo Horizonte, Minas Gerais 30130100, Brazil; 2 Graefe Institute of Ophthalmology, Rua Capitão Souza Franco, 95, 80035-050, Curitiba, Parana, Brazil; 3 Veterinary Department, Federal University of Parana, Rua dos Funcionarios 1540, 80035-050, Curitiba, Brazil; 4 Pathology Department, Pontifical Catholic University of Parana, Rua Imaculada Conceição 1155, Prado Velho CEP, 80215-901, Curitiba, Parana, Brazil; 5 Ophthalmology Department, Federal University of São Paulo, Rua Botucatu, 821, 2nd floor, 04023-062, Sao Paulo, Sao Paulo, Brazil

**Keywords:** Glaucoma, Prostaglandin analogues, Timolol, Conjunctiva, Histology, Immunohistochemistry

## Abstract

**Background:**

The purpose of this research is to compare the histological and immunohistochemical changes induced by fixed combinations of timolol maleate and prostaglandin analogues in the rabbit conjunctiva. Thirty left eyes of rabbits, divided into three groups, were treated for 30 days with the following combinations of drugs: bimatoprost 0.03% + timolol 0.5%, travoprost 0.004% + timolol 0.5% and latanoprost 0.005 + timolol 0.5%. The right eyes served as controls and received no medication. At the end of the experiment, after enucleation, the conjunctivas were assessed through histomorphometry (number of inflammatory and goblet cells, epithelial thickness) and immunohistochemistry (anti-actin antibody to assess the degree of fibrosis).

**Results:**

Histomorphometrically, there was infiltration of inflammatory cells in all the treated eyes. An increased number of goblet cells was observed with the use of all fixed combinations of prostaglandin analogues associated with timolol maleate in comparison with the control group. The combination travoprost + timolol resulted in more intense fibrosis. The effect of bimatoprost + timolol caused an intermediate reaction pattern among the other drugs, fostering higher numbers of goblet cells in the conjunctival epithelium, more than the other fixed combinations in this study. There was a difference in the comparison of goblet cells of eyes treated with bimatoprost + timolol (16.11 ± 2.42) and of those treated with latanoprost + timolol (13.18 ± 1.60) (*P* = 0.016).

**Conclusion:**

It was found that all fixed combinations of prostaglandins analogues + timolol induce a reaction in the conjunctiva, increasing the inflammatory infiltrate.

## Background

Glaucoma is an optic neuropathy, which is progressive, chronic, multifactorial and often associated with increased intraocular pressure, and requires long-term treatment with topical hypotensive medication [1]. Several classes of drugs are currently available for treating glaucoma, including cholinergic agents, beta blockers, alpha adrenergic agonists, carbonic anhydrase inhibitors, and more recently prostaglandins analogues (PG) and the fixed combinations (FC) of alpha adrenergic agonists, carbonic anhydrase inhibitors or prostaglandin analogues associated with timolol 0.5% [2]. PG analogues increase the uveoscleral outflow and can reduce intraocular pressure up to 40% of its initial value [3-5]. Systemic secondary side effects induced by topical PG analogues are rare. Local secondary side effects caused by these drugs are thus reported: conjunctival hyperemia that can be seen in 40% of patients [6], double the amount of what is found in patients using timolol [7], and in addition to iris hyperpigmentation and periorbicular hyperpigmentation, excessive growth of eyelashes [8-10]. In patients treated with topical medications, the conjunctiva acts as a semipermeable membrane that, together with the cornea, enables the absorption of ocular hypotensors [11]. Moreover, the conjunctiva responds to the chronic antiglaucoma treatment drug with inflammation, scarring, keratinization and neovascularization, which may directly affect its architecture and function [12]. Some studies have shown that the main topical medications used to treat glaucoma increase the number of fibroblasts and inflammatory cells in the conjunctival substantia propria and induce epithelial metaplasia, causing changes in its structure. High concentrations of macrophages, lymphocytes, mast cells and fibroblasts, in addition to the reduction of goblet cell density, were reported in the conjunctiva in patients having chronic treatment with beta blockers [13]. These cellular changes can cause tear film instability, leaving the cornea and conjunctiva more exposed to external agents [14]. Some studies describe the conjunctival changes related to the use of topical antiglaucomatous drugs; however, little is known about the effects of conjunctival prostaglandin analogues [15]. Most research were conducted with latanoprost, but there is still insufficient information on the effects of other newer analogues such as bimatoprost and travoprost [4,16]. This study aimed to evaluate the structural and inflammatory changes induced by fixed combinations of prostaglandin analogues (latanoprost, travoprost and bimatoprost) and timolol maleate.

## Methods

Thirty New Zealand female rabbits, on average 4 months old and weighing 1.5 kg, were divided into three groups of ten. For 30 days, the left eye of each animal received one drop of eye solution with the fixed combination of timolol 0.5% associated with one of the following prostaglandin analogues at 5 pm: group 1 received bimatoprost 0.03%; group 2, travoprost 0.004%; group 3, latanoprost 0.005%. The right eyes served as controls and received no medication. The assessment was conducted within 30 days, as in previous studies, and observed changes in the conjunctiva and the use of immunohistochemistry showed higher accuracy and evaluated data very early. After 30 days, all rabbits were euthanized, and subsequent bilateral enucleation was performed. The eyes were immediately treated with 10% formaldehyde for 24 h. The superior limbus was marked before processing because we tried to assess the location of a possible filtering surgery and to compare similar areas in two eyes; because there may be a change according to the location, there should be less depot medications, nasal and temporal greatest environmental exposure and keratinization. Next, eyes were sectioned and passed through pattern processing dehydration with increasing degrees of alcohol, up to 90% absolute alcohol, and subsequent clarification with xylene, embedded in paraffin [17]. Microtome was used for sectioning the paraffin block stained with hematoxylin-eosin (HE) and periodic acid-Schiff (PAS) and sustained immunohistochemistry (IHC) with the following label: anti-actin (ICN Biochemicals, Irvine, CA, USA; dilution 1:10) blades. Specimens stained with HE, PAS and anti-actin were photographed under ×400 magnification and analysed with the Image-Pro Plus 4.5 software (Media Cybernetics, Silver Spring, MD, USA). Points of each blade were chosen, corresponding to a conjunctival flap closest to the limbus. Each image, including the epithelium and lamina propria, was virtually barred (150 × 200 mm), and the three subfields closer to the epithelium were then evaluated to enable a more specific analysis of the subepithelial region of the conjunctiva, and not of its lamina propria in each slide.

The following parameters were histomorphometrically evaluated: number of inflammatory cells (HE), epithelial thickness (HE) and number of goblet cells (PAS). In IHC, the following was observed: qualitative assessment of the degree of fibrosis (fibroblasts marked by actin), which was classified as minimum [1], mild [2], moderate [3] and severe [4]. The statistics of numerical variables was performed with the one-way ANOVA test with a significance level of 5%, followed by the post-hoc Tukey-Kramer test. The evaluation of ordinal categorical variables (degree of fibrosis marker in anti-actin) was performed using analysis of descriptive statistics and Fisher's exact test. The experimental procedures were approved by the Ethics Committee on Animal Use of the Department of Agrarian Sciences, Federal University of Parana (CEUA in SCA 038/2008).

## Results

**Figure 1:**
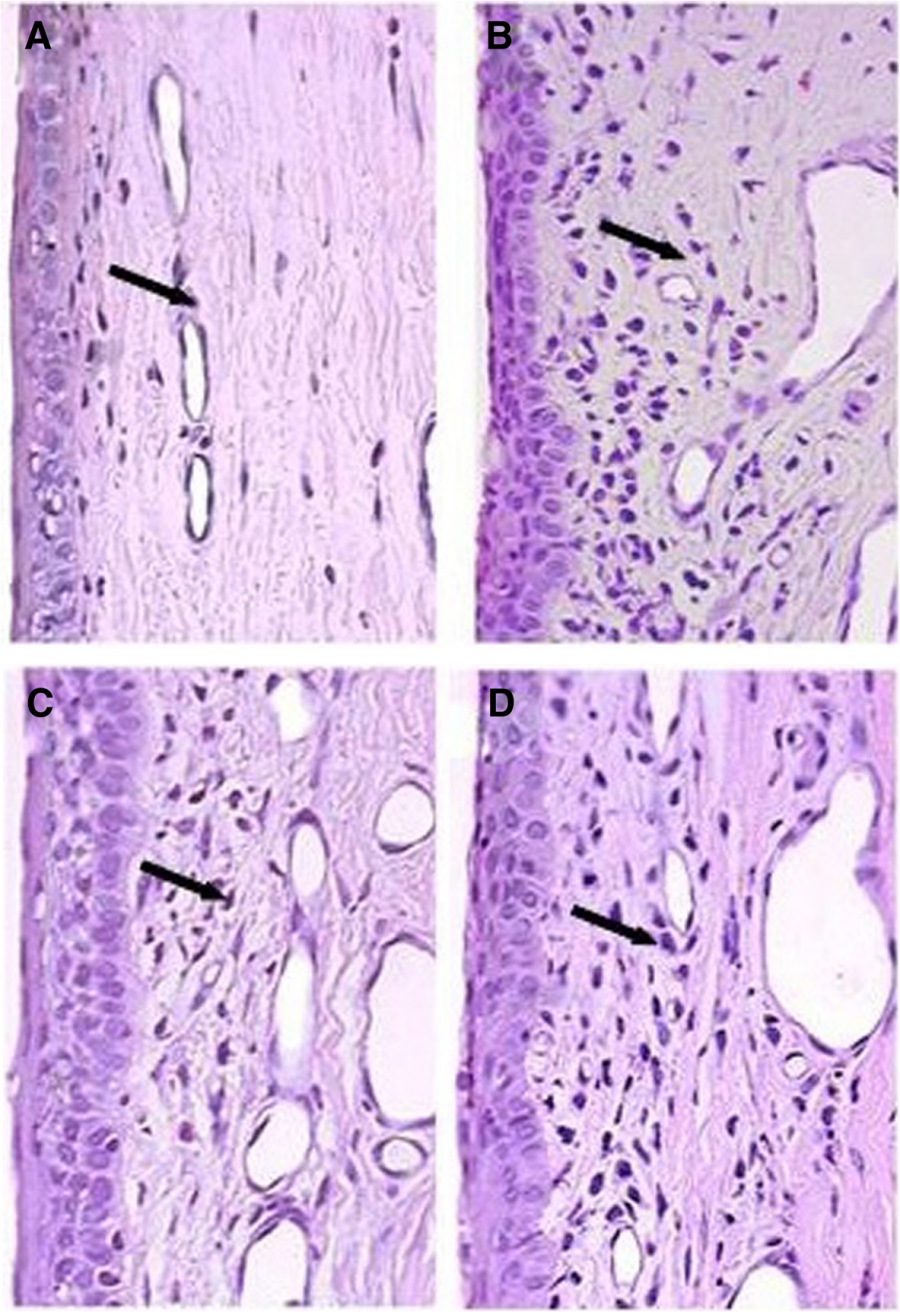
**Photomicrographs of conjunctiva (magnification of ×400) stained with HE. **The photomicrographs show the increase of inflammatory cells in eyes treated with (**B**) bimatoprost + timolol, (**C**) travoprost + timolol and (**D**) e latanoprost + timolol, compared to (**A**) the control eye.

**Figure 2:**
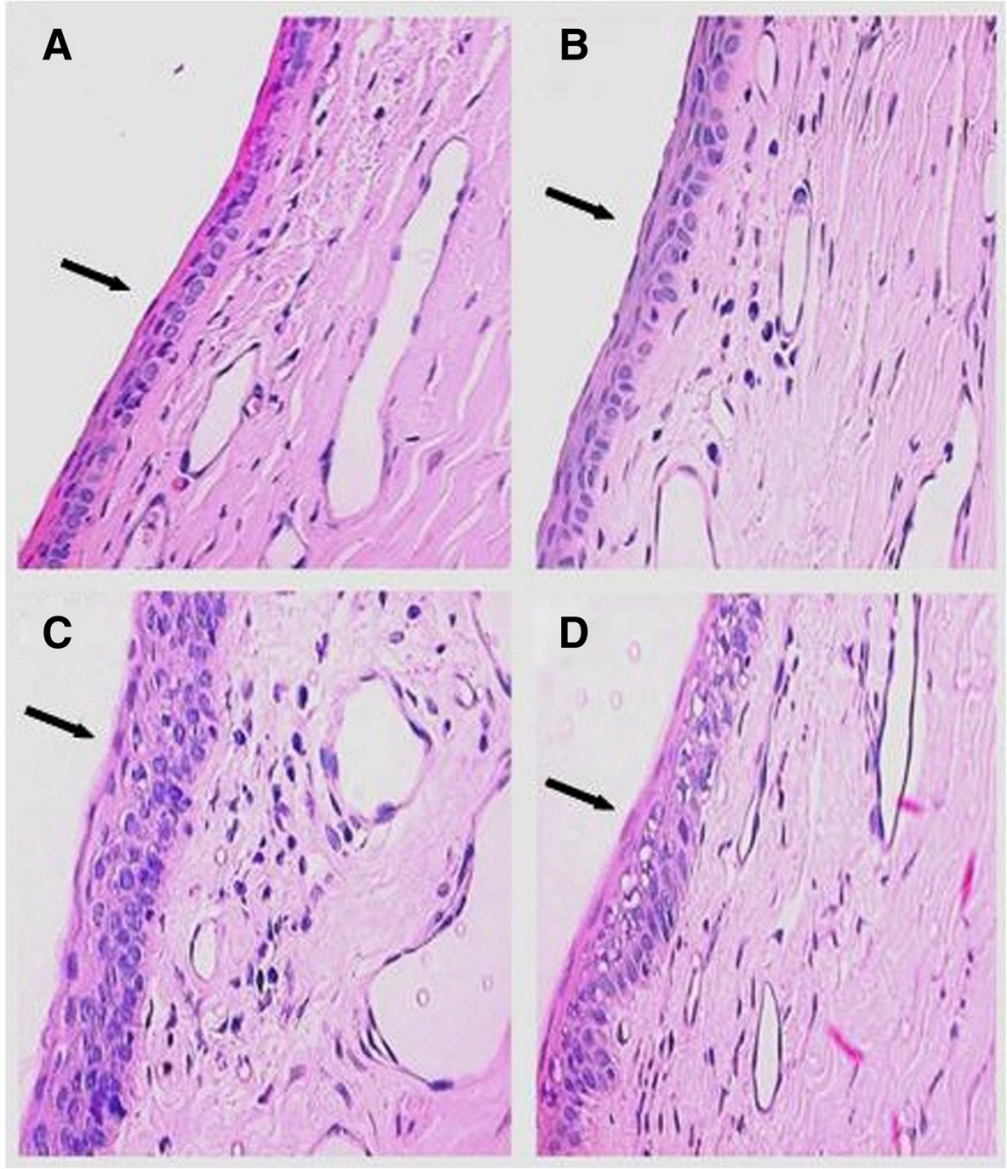
**Photomicrographs of conjunctiva (magnification of ×400) stained with HE. **The photomicrographs show the increase in the epithelial thickness in eyes treated with (**B**) bimatoprost + timolol, (**C**) latanoprost + timolol and (**D**) e travoprost + timolol, compared to (**A**) the control eye. The largest thickness was observed in the group treated with latanoprost + timolol.

An increase was observed in the number of conjunctival inflammatory cells in all groups when compared to the control eyes. There was no significant difference among groups of treated eyes (Table 1; Figures 1 and 2).

**Table 1:** Results of parameters in groups of fixed combinations: inflammatory cells, epithelial thickness and goblet cells

**Parameters**	**Stain**	**Bimatoprost + timolol**					**Travoprost + timolol**					**Latanoprost + timolol**				
		**Control**		**Treated**		***P***	**Control**		**Treated**		***P***	**Control**		**Treated**		***P***
		**Mean**	**SD**	**Mean**	**SD**		**Mean**	**SD**	**Mean**	**SD**		**Mean**	**SD**	**Mean**	**SD**	
Inflammatory cells	HE	17.44	5.03	34.4	10.12	0.0003	17.11	3.1	33.33	6.26	0.0001	18.54	3.64	30.72	5.97	0.0001
Epithelial thickness	HE	19.56	1.97	22.24	3.02	0.0305	18.75	2.08	23.88	3.94	0.0032	19.44	2.83	26.55	4.75	0.0004
Goblet cells	PAS	8.6	1.17	16.11	2.42	0.0001	8	0.71	14.44	1.42	0.0001	7.9	1.37	13.18	1.6	0.0001

The conjunctival epithelial thickness is also increased in all treated eyes. This increase was more intense in the group treated with latanoprost + timolol (19.44 ± 2.83 to 26.55 ± 4.75; *P* = 0.0004), followed by the group treated with travoprost + timolol (18.74 ± 2.08 to 23.88 ± 3.94; *P* = 0.0032) and finally that treated with bimatoprost + timolol (19.56 ± 1.97 to 22.24 ± 3.02; *P* = 0.0305) (Graphic 1 in Additional file 1; Figure 2).

**Figure 3:**
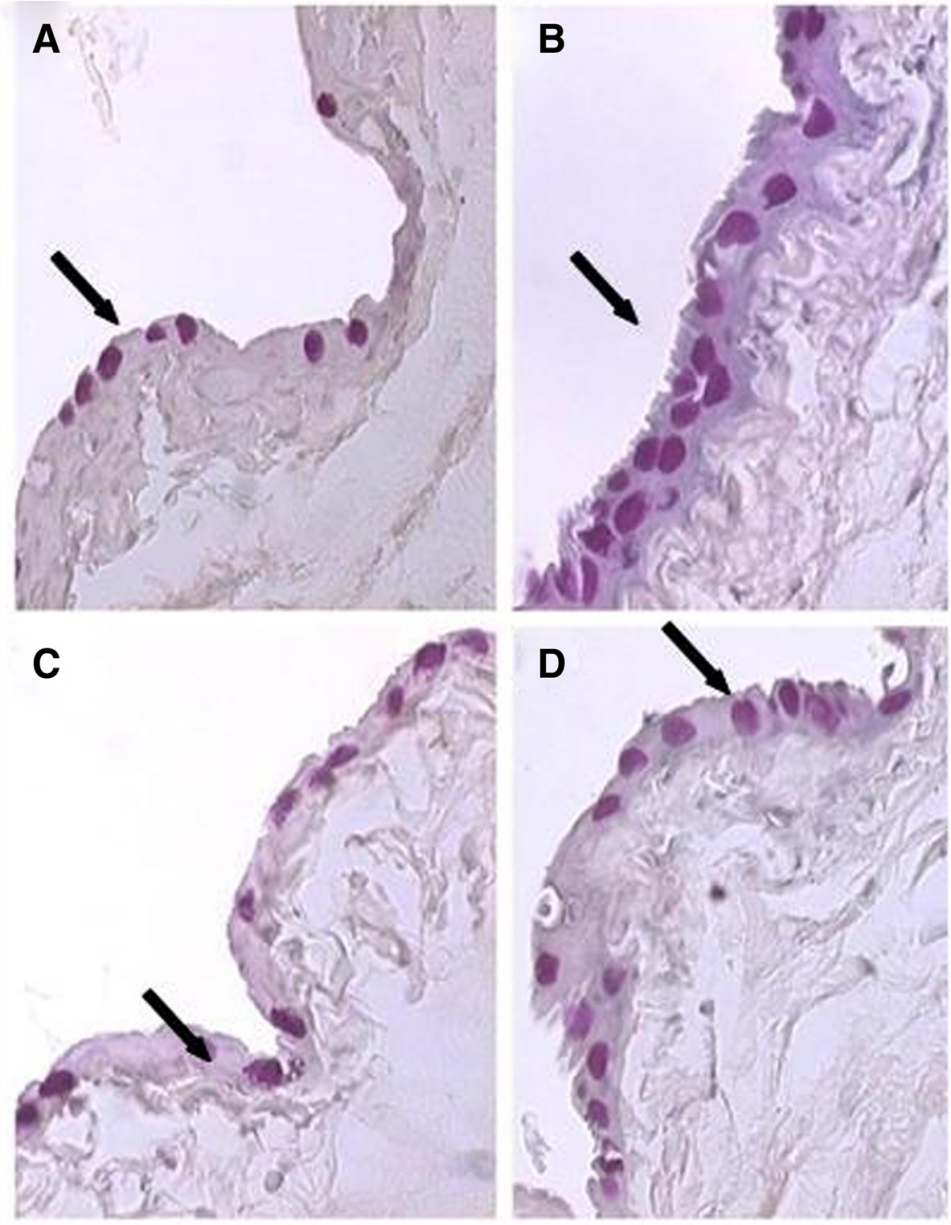
**Photomicrographs of conjunctiva (magnification of ×400) stained with PAS. **The photomicrographs show the higher increase in the number of goblet cells in eyes treated with (**B**) bimatoprost + timolol and (**D**) travoprost+timolol and the lower increase in eyes treated with (**C**) latanoprost + timolol, compared to (**A**) the control eye.

The number of goblet cells in treated eyes increased as compared to control eyes in all groups. There was a noticeable difference in eyes treated with bimatoprost + timolol (16.11 ± 2.42) and those treated with latanoprost + timolol (13.18 ± 1.60; *P* = 0.016) (Figure 3).

**Figure 4:**
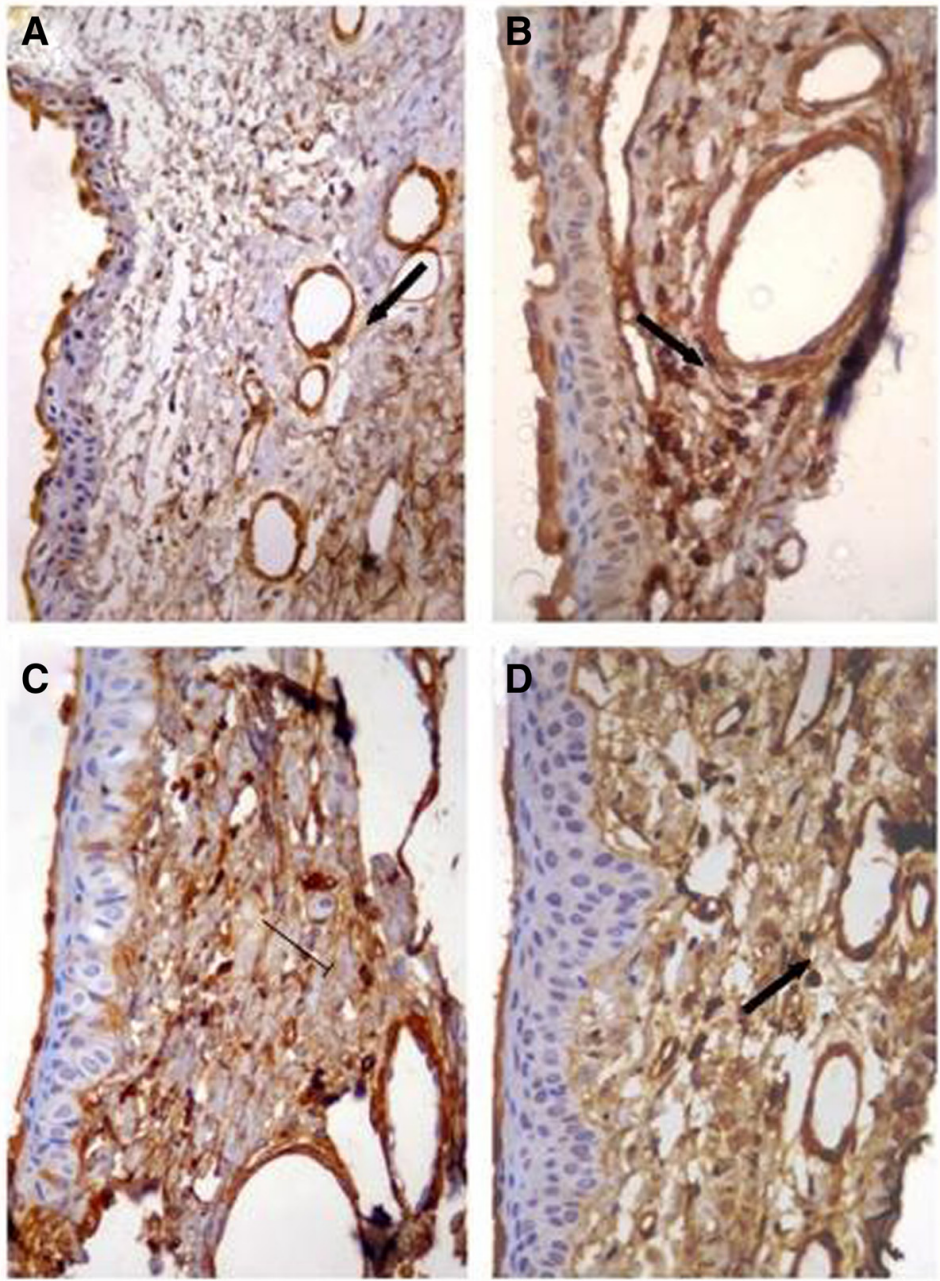
**Photomicrographs of conjunctiva (magnification of ×400) stained with anti-actin. **The photomicrographs show the increase in number of fibroblasts in eyes treated with (**B**) travoprost + timolol, (**C**) latanoprost + timolol and (**D**) bimatoprost + timolol, compared to (**A**) the control eye.

The conjunctiva of all eyes (treated and control groups) stained with anti-actin demonstrated some degree of fibrosis. In the control eyes, this degree ranged from minimal to mild. In eyes treated with timolol + travoprost, a greater degree of fibrosis was observed (median, 3), followed by the bimatoprost + timolol group (median, 2.5) and then the latanoprost + timolol group (median, 2). These differences, while displaying a numerical trend, were not statistically significant (Graphic 2 in Additional file 1; Figure 4).

## Discussion

Treatment with topical antiglaucoma drugs has been associated with ocular surface disease, which includes an increase in the subepithelial collagen deposition, reduction of the number of goblet cells and increased number of mast cells, fibroblasts, lymphocytes and macrophages in the substantia itself. In addition to the effects of conjunctival changes, a delay in the corneal epithelial regeneration and tear film instability due to a reduction in the portion of tear mucus occur. These changes cause discomfort at instillation of eye drops and become a potential risk of filtering failure in surgical procedures such as trabeculectomy [11,14,18]. A study by Baudouin et al. [13] which evaluated the conjunctiva, using biopsy of patients treated with long-term use of antiglaucoma drugs, found a high concentration of macrophages, lymphocytes, mast cells and fibroblasts besides a reduction in the number of goblet cells.

In this study, an increase in the number of inflammatory cells was noticed in eyes treated with a fixed combination of timolol maleate and prostaglandin analogues. However, there also was an increased number of goblet cells in all groups (latanoprost + timolol, travoprost + timolol and bimatoprost + timolol). This increase is probably associated with the prostaglandin analogues, and not to timolol, since previous studies have shown that beta blockers induce a reduction in the number of goblet cells and tear production [19]. Russ et al. [16] also observed an increase in the conjunctival goblet cells in rabbits treated with prostaglandin analogues, which was not observed in those treated with the timolol.

Another fact to be considered is the result of a study that showed that goblet cell hyperplasia of the conjunctiva is associated with polluted environments, indicating that this increase may be an ocular response to external aggression [20]. The increase in goblet cells caused by prostaglandin analogues can be associated with a protective effect of the ocular surface but could also be a reflection of the higher capacity for external aggression of these drugs. Russ et al. [16] observed a less severe inflammatory infiltrate in groups treated with prostaglandin analogues than in the group treated with timolol. Our research showed a significant increase in the number of inflammatory cells in all treated eyes, but there was no difference in the degree of inflammatory reaction incited by comparing the three combinations. This increase is probably more related to timolol than the prostaglandin analogues. Another factor to be considered is the presence of benzalkonium chloride (BAK) in such formulations. BAK is the most commonly used preservative in antiglaucoma drugs [14]. Many studies have related the effect of cell toxicity and inflammation of the ocular surface caused by topical ophthalmic therapy with BAK [21]. Comparisons between use of non-preserved timolol and timolol preserved with BAK showed a lower level of toxic effects in the first group and induction of inflammatory infiltrate in the group treated with the preservative [13]. *In vitro* studies showed that none of prostaglandin analogues appear to induce direct stimulation of inflammatory pathways involving adhesion molecules or class II antigens. This pattern of toxicity seems to be associated with BAK. Latanoprost and travoprost have been considered as responsible for the protective effects against BAK toxicity, possibly related to antioxidant properties [15]. This protective inflammatory effect was proposed by Guenoun et al. [22], who noted, *in vitro*, that prostaglandins tended to be less toxic when compared to BAK. Cvenkel and Ihan [23] carried out flow cytometry to determine the levels of HLA-DR (molecules expressed by macrophages, B lymphocytes and activated T lymphocytes) in patients' imprint cytology conjunctival samples without clinical signs of inflammation, treated with antiglaucomatous drugs (latanoprost, betaxolol and timolol) containing BAK preservative. It was noticed that all treated eyes, regardless of the drug applied, showed increased expression of HLA-DR, which indicates that topical therapy with BAK induces subclinical inflammation of the conjunctiva. Broadway and colleagues [11] studied the ocular changes in patients treated for more than 3 years with antiglaucomatous drugs, isolated or in combination (beta blockers, miotics and sympathomimetics). These studies have shown a reduction in the number of goblet cells, an increased number of macrophages and lymphocytes in the conjunctival epithelium and increased number of fibroblasts, macrophages, mast cells and lymphocytes in the substantia itself, as well as epithelial metaplasia with alterations in the epithelium structure. The compared treatments in this study showed that the conjunctiva was affected in different ways, the main effect occurring in protocols associated to more than one drug. The increased epithelial thickness observed is consistent with results previously reported in literature.

In an experiment conducted in rabbits by Mietz et al. [18], it was noted that eyes treated with preserved timolol were stained with anti-actin antibody, an antibody directed to the proteins of the cellular cytoskeleton of fibroblasts. In our study, the subepithelium of the treated eyes of all groups was stained with the same antibody. There was no significant difference between groups, but a numerical trend with a more intense degree of reaction in the group treated with travoprost + timolol was noticed.

## Conclusion

The evaluation of the inflammatory infiltrate in the treated eyes, in this study, showed no difference in the number of inflammatory cells, comparing the three fixed combinations. The fixed combination travoprost + timolol showed a higher degree of fibrosis. The fixed combination of bimatoprost + timolol led to a greater increase in the number of goblet cells.

## Competing interests

The authors declare that they have no competing interests.

## Authors’ contribution

NVF, HHR and FMF designed the study. NVF and PR conducted the study. Analysis and interpretation of the data were done by NVL, HHR, SCS and FM. NVF, PR and HHR wrote the article. Critical revision of the article was done by HHR and SCS; NVF, HHR, SCS and PAM gave the final approval of the article. NVF and PR provided the materials and resources, FMF lent his statistical expertise, HHR obtained funds, NVR and PR performed the literature search and LN provided administrative, technical and logistic and support. All authors read and approved the final manuscript.

## Supplementary Material

Additional file 1 **Graphic 1. **shows the epithelial thickness of rabbit conjunctiva (HE), comparing control to treated eyes in the three groups: bimatoprost + timolol (G1), travoprost + timolol (G2) and latanoprost + timolol (G3). Observe the increase in epithelial thickness in all treated eyes, comparing to their respective controls, especially in latanoprost + timolol. Histological evaluation performed with HE.
**Graphic 2. **shows the degree of conjunctival fibrosis of rabbit comparing the controls with the treated eyes in the three groups: bimatoprost + timolol (G1), travoprost + timolol (G2) and latanoprost + timolol (G3). A higher degree of fibrosis was observed in eyes treated with travoprost + timolol. Immunohistochemical evaluation performed with anti-actin. Click here for file
