# Supplementary material for: Conjunctival changes and inflammatory aspects in rabbits' conjunctivas induced by fixed combinations of prostaglandin analogues and timolol maleate
Source: J Ophthalmic Inflamm Infect. 2013 Jan 28;3:22. doi: 10.1186/1869-5760-3-22 (PMC3605097; doi:10.1186/1869-5760-3-22)
Supplement: Additional file 1 — Graphic 1. shows the epithelial thickness of rabbit conjunctiva (HE), comparing control to treated eyes in the three groups: bimatoprost + timolol (G1), travoprost + timolol (G2) and latanoprost + timolol (G3). Observe the increase in epithelial thickness in all treated eyes, comparing to their respective controls, especially in latanoprost + timolol. Histological evaluation performed with HE. Graphic 2. shows the degree of conjunctival fibrosis of rabbit comparing the controls with the treated eyes in the three groups: bimatoprost + timolol (G1), travoprost + timolol (G2) and latanoprost + timolol (G3). A higher degree of fibrosis was observed in eyes treated with travoprost + timolol. Immunohistochemical evaluation performed with anti-actin. [file 1869-5760-3-22-S1.doc]

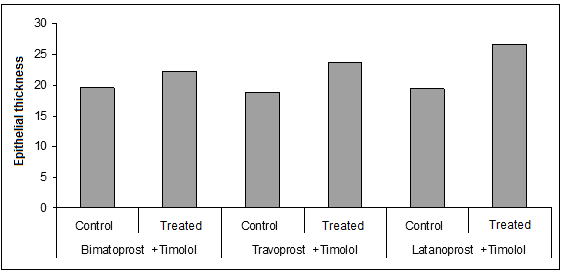


Graphic 1 – Epithelial thickness of rabbit conjunctiva (HE), comparing control to treated eyes in the three groups: bimatoprost+timolol (G1), travoprost+timolol (G2) and latanoprost+timolol (G3). Observe the increase in epithelial thickness in all treated eyes, comparing to their respective controls, especially in latanoprost+timolol. Histological evaluation performed with HE.


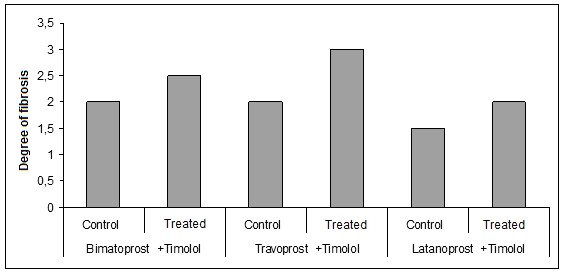


Graphic 2– Degree of conjunctival fibrosis of rabbit comparing the controls with the treated eyes in the three groups: bimatoprost+timolol (G1), travoprost+timolol (G2) and latanoprost+timolol (G3). A higher degree of fibrosis was observed in eyes treated with travoprost+timolol. Immunohistochemical evaluation performed with anti-actin.
